# Esterase-Responsive Polyglycerol-Based Nanogels for Intracellular Drug Delivery in Rare Gastrointestinal Stromal Tumors

**DOI:** 10.3390/ph16111618

**Published:** 2023-11-16

**Authors:** Sebastian Schötz, Adele K. Griepe, Björn B. Goerisch, Sally Kortam, Yael Shammai Vainer, Mathias Dimde, Hanna Koeppe, Stefanie Wedepohl, Elisa Quaas, Katharina Achazi, Avi Schroeder, Rainer Haag

**Affiliations:** 1 Institute of Chemistry and Biochemistry, Freie Universität Berlin, Takustr, 3, 14195 Berlin, Germany; bastischoetz@zedat.fu-berlin.de (S.S.); adeg98@zedat.fu-berlin.de (A.K.G.); bjoernbog00@zedat.fu-berlin.de (B.B.G.); hanna.koeppe@fu-berlin.de (H.K.); 2 School of Biomedical Engineering, The University of Sydney, Sydney, NSW 2006, Australia; skur9478@uni.sydney.edu.au; 3 The Louis Family Laboratory for Targeted Drug Delivery and Personalized Medicine Technologies, Technion, Haifa 32000, Israel; yael.shammai@campus.technion.as.il; 4 Research Center of Electron Microscopy, Freie Universität Berlin, Fabeckstr, 36A, 14195 Berlin, Germany; mathias.dimde@fu-berlin.de; 5 Research Building SupraFAB, Freie Universität Berlin, Altensteinstr, 23a, 14195 Berlin, Germanyequaas@zedat.fu-berlin.de (E.Q.); katharina.achazi@fu-berlin.de (K.A.)

**Keywords:** nanogels, drug delivery, GIST, iEDDA, polyglycerol

## Abstract

Rare gastrointestinal stromal tumors (GISTs) are caused by mutations in the KIT and PDGFRA genes. Avapritinib (BLU-285) is a targeted selective inhibitor for mutated KIT and PDGFRA receptors that can be used to treat these tumors. However, there are subtypes of GISTs that exhibit resistance against BLU-285 and thus require other treatment strategies. This can be addressed by employing a drug delivery system that transports a combination of drugs with distinct cell targets. In this work, we present the synthesis of esterase-responsive polyglycerol-based nanogels (NGs) to overcome drug resistance in rare GISTs. Using inverse nanoprecipitation mediated with inverse electron-demand Diels–Alder cyclizations (iEDDA) between dPG-methyl tetrazine and dPG-norbornene, multi-drug-loaded NGs were formed based on a surfactant-free encapsulation protocol. The obtained NGs displayed great stability in the presence of fetal bovine serum (FBS) and did not trigger hemolysis in red blood cells over a period of 24 h. Exposing the NGs to Candida Antarctica Lipase B (CALB) led to the degradation of the NG network, indicating the capability of targeted drug release. The bioactivity of the loaded NGs was tested in vitro on various cell lines of the GIST-T1 family, which exhibit different drug resistances. Cell internalization with comparable uptake kinetics of the NGs could be confirmed by confocal laser scanning microscopy (CLSM) and flow cytometry for all cell lines. Cell viability and live cell imaging studies revealed that the loaded NGs are capable of intracellular drug release by showing similar IC_50_ values to those of the free drugs. Furthermore, multi-drug-loaded NGs were capable of overcoming BLU-285 resistance in T1-α-D842V + G680R cells, demonstrating the utility of this carrier system.

## 1. Introduction

The most frequent mesenchymal neoplasm of the digestive system are gastrointestinal stromal tumors (GISTs). GISTs are believed to develop from the interstitial cells of Cajal or related stem cells. They are classified by proto-oncogene receptor tyrosine kinase KIT or platelet-derived growth factor receptor alpha (PDGFRA)-activating mutations [1,2]. Approximately 80% of GISTs contain KIT gene mutations, while an additional 10% display PDGFRA gene mutations, resulting in constitutive activation of the KIT receptor and downstream signaling pathways that promote cell survival, growth, and proliferation [3,4,5]. GISTs occur most frequently in the stomach (60%) and small intestine (25%). However, they can also be found in the rectum (5%), esophagus (2%), and several other places (5%), including the appendix, gallbladder, pancreas, mesentery, omentum, and retroperitoneum [6,7,8,9,10,11,12,13,14,15]. Surgical resection is the most common form of GIST treatment. Patients with low-risk or intermediate-risk cancers have reasonably favorable outcomes, whereas recurrence is nearly unavoidable after the excision of high-risk tumors [16]. Single-agent and combination chemotherapy trials have routinely failed to produce partial response rates of more than 5% [17]. These unfavorable observations could be attributed to high levels of multi-drug resistance protein expression in many GISTs [18,19].

With the development of novel medications, such as Sunitinib, Imatinib, and Avapritinib (BLU-285), which target the cancer-specific KIT and PDGFRA tyrosine kinase receptors, the previously bleak prospects for patients with locally progressed or metastatic GISTs have significantly improved. Secondary genetic mutations, on the other hand, induce drug resistance and impede the development of new therapeutic options. For example, GIST cells with the common D842V mutation in the PDGFRA gene are relatively insensitive to the drug Imatinib [20]. Furthermore, patients with the D842V mutation might generate a secondary mutation, D842V + G680R, which makes them resistant to BLU-285 [4]. Using a combination of drugs is one technique for combating drug resistance in GISTs. Tanespymicin (17-AAG) is a geldanamycin derivative that preserves significant anticancer action while reducing hepatotoxicity and improving bioavailability [21]. It binds specifically to the ATP-binding domain of heat shock protein 90 (HSP 90), preventing the development of the HSP 90 multichaperone complex. This causes client proteins to be degraded via the ubiquitin–proteasome pathway [22]. Even though HSP 90 is present in normal cells, its overexpression has been linked to the development of many solid tumors. As a result, it has been proposed as a universal biomarker for cancer progression. According to research, 17-AAG can selectively target cancer cells and restrict tumor growth [23]. Furthermore, it was shown that combining imatinib with 17-AAG had a synergistic effect and could be used to address imatinib-resistant cells [24]. However, due to 17-AAG’s poor water solubility, systemic administration requires drug solubilizers, such as DMSO. Moreover, 17-AAG has a short half-life circulation time and reasonable hepatotoxicity, limiting its clinical application [22]. Hydrophobic drugs can be encapsulated non-covalently to circumvent these difficulties in nano-sized drug delivery systems, such as nanogels (NGs). NGs are crosslinked, three-dimensional networks of hydrophilic or amphiphilic polymers with sizes ranging from 10 to 1000 nm [25,26]. They can be manufactured from hydrophilic polymers that swell in aqueous media, such as polyethylene glycol [27], polyoxazoline [28], dendritic polyglycerol (dPG) [29], and hyaluronic acid [30]. They have a variety of adjustable characteristics that can be used to improve drug loading, specify drug release, and target specific cell receptors. Thereby, a common strategy is the incorporation of degradable bonds that can be addressed by disease-specific stimuli such as the overexpression of esterases and lipases [31], reduced intracellular pH values in endo- and lysosomes [32], and elevated levels of GSH in the cytosol of cells [33].

Mini- and micro-emulsion polymerizations are routinely used to produce NGs. However, this approach necessitates the use of surfactants, and removing them after the reaction has ended can become challenging. As an alternative, Steinhilber et al. proposed a surfactant-free preparation process based on adding a macromonomer solution into a non-solvent while vigorously stirring. This results in the formation of nano-sized aggregates in which the particles crosslink [34]. Despite its mild and surfactant-free conditions, this approach demands crosslinking strategies with fast reaction kinetics that can be carried out in an aqueous environment and with crosslinking moieties that are inert to other functional groups. This can be addressed by click-type reactions, such as the inverse electron-demand Diels–Alder cycloaddition (iEDDA), between methyl tetrazines and norbornenes.

In this work, we report the synthesis of esterase-responsive dPG-based NGs that can address drug resistance in rare GISTs. NGs were formed through iEDDA-mediated nanoprecipitation. Loading of the NGs with BLU-285 and 17-AAG was achieved through co-precipitation of the drugs. Applying the enzyme Candida Antarctica Lipase B (CALB) to the NGs initiated network degradation. In vitro experiments were performed to investigate the NGs’ compatibility with fetal bovine serum (FBS) and red blood cells and the system’s ability to treat multiple GIST cells with varying drug resistances.

## 2. Results

### 2.1. Macromonomer Synthesis

**Scheme 1:**
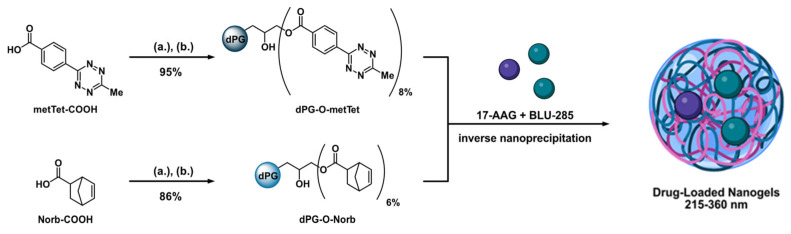
Synthetic overview of NG formation from precursors. (a.) DMAP, EDC·HCl, HATU, DMF, rt, 60 min. (b.) dPG (10 kDa), DMAP, pyridine, 60 °C, o.n.

dPG belongs to the polymer class of polyethers and displays excellent stealthing properties and biocompatibility [35,36,37]. However, it lacks inherent biodegradability, limiting its utilization to smaller-sized polymer fragments below the renal threshold to prevent accumulation in unwanted organs and undesired tissues. These macromonomers can be crosslinked with biodegradable linkers to obtain high molecular weight dPGs. The overall strategy in this work was to develop esterase-responsive dPG-based NGs through iEDDA crosslinking. dPG was synthesized in an anionic ring-opening polymerization starting from glycidol. Under Steglich esterification conditions, further functionalization of the exterior hydroxyl groups with Norb-COOH and metTet-COOH resulted in the formation of dPG-O-metTet and dPG-O-Norb with 96% yield and 86% yield, respectively. The occurring iEDDA between both macromonomers during inverse nanoprecipitation led to the formation of NGs (Scheme 1).

### 2.2. Nanogel Formation and Drug Loading

**Figure 1:**
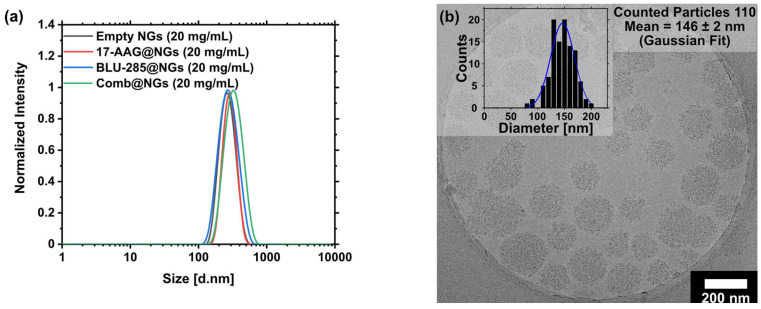
Results of NG formation. (**a**) Size distribution of loaded and empty NGs determined by DLS. (**b**) cryo-TEM of free NGs in PBS at 1.00 mg/mL, showing the spherical appearance of the particles. Gaussian analysis of multiple measurements determined the particle size.

Inverse nanoprecipitation was performed by premixing an aqueous solution of dPG-O-metTet and dPG-O-Norb, followed by their subsequent injection into acetone. Crosslinking was terminated by the addition of water. This method relies on the addition of a good solvent, such as water, into a non-solvent, such as acetone. Due to a reduction in the precursors’ solvent quality, their homogenous nucleation in nano-sized water droplets occurs, followed by iEDDA-mediated crosslinking. NGs were obtained after evaporation of the organic solvent under reduced pressure as a stable dispersion in water. Their size and spherical morphology were proven by dynamic light scattering (DLS) and cryogenic transmission electron microscopy (cryo-TEM). Gaussian analysis of the detected particles in cryo-TEM revealed a mean size of 146 nm, slightly smaller than the measured hydrodynamic diameter of 257 nm in DLS. This can be explained by the hydration shell that forms around particles during DLS analysis but is absent during cryo-TEM measurements (Figure 1a,b). Co-precipitation of 17-AAG and BLU-285 was achieved by premixing the drugs with one of the precursors and led to the formation of multi-drug-loaded NGs with hydrodynamic diameters ranging from 250 to 360 nm. Loading experiments were carried out with a drug loading ratio of 5 wt%. Quantification of the obtained drug loading contents (DLCs) was determined by UV/Vis spectroscopy for 17-AAG using its unique absorbance at 550 nm (Table 1, Figure S1) and by fluorescence spectroscopy for BLU-285 using 380 nm as the excitation wavelength and 440 nm as the absorbance wavelength (Table 1, Figure S2). For the single-drug-loaded NGs (17-AAG@NGs and BLU-285@NGs), a DLC of 4.95 wt% could be determined, yielding encapsulation efficiencies (ee) of 99%. For combinational loading, quantitative ee could be obtained for 17-AAG, and slightly reduced DLCs of 3.10 wt% could be observed for BLU-285, indicating saturation of the system. These experiments suggest that the NGs are capable of encapsulating hydrophobic drugs in their polymer network. Drug retainment is most likely achieved through hydrophobic interaction between the cargo and the organic linker structure of the reacted tetrazine and norbornene moieties.

### 2.3. Nanogel Degradation

**Figure 2:**
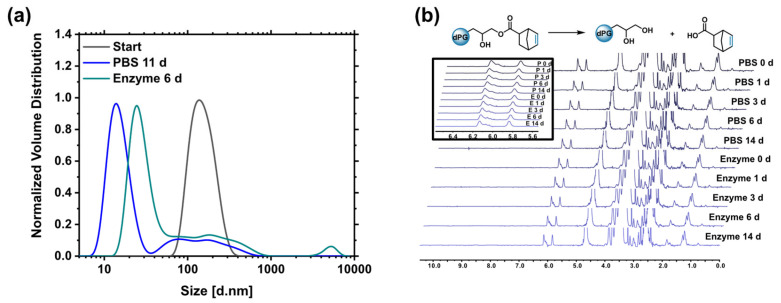
Overview of degradation experiments. (**a**) Degradation of NGs in the presence of PBS (blue) and Novozyme-435 (cyan) determined by DLS. (**b**) Degradation of dPG-O-Norb in the presence of PBS (top) and Novozyme-435 (down) determined by ^1^H NMR measurements. The insert shows the signal of the double bond of norbornene, indicating that NG degradation can be related to ester hydrolysis.

To investigate the susceptibility of the present ester bonds, NGs were exposed to CALB and incubated under physiological conditions (PBS 154 mM, pH 7.4, 37 °C). DLS measurements of the corresponding NGs revealed a shift of the hydrodynamic diameter to smaller-sized values of about 20 nm after 6 days of enzyme incubation. Similar changes could be observed for the PBS control after 11 days of incubation, indicating an ester response to CALB (Figure 2a). As proof, ^1^H NMR degradation experiments were carried out with the dPG-O-Norb macromonomer. The shift in the proton signal of the norbornene double bond was used to examine the degradation. Similar to the DLS experiment, the esters responded faster to CALB since the appearance of the reference peak began to change after 3 days for CALB and 14 days for the PBS control (Figure 2b).

### 2.4. Compatibility Studies

**Figure 3:**
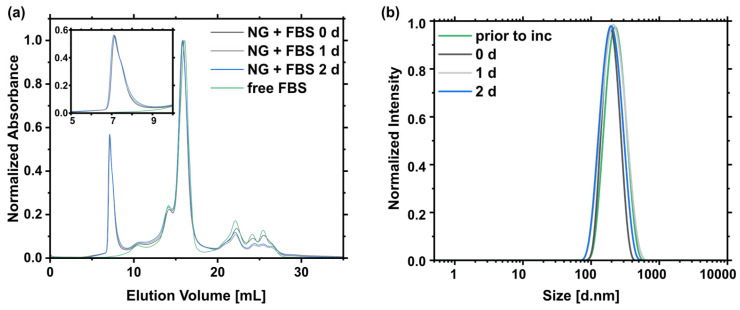
Results of FBS stability studies. (**a**) Chromatograms of NG + FBS. The insert shows the peak that is referring to the NG. (**b**) Size distribution determined by DLS of isolated NGs after FBS incubation.

The biocompatibility of the NGs was investigated by performing stability studies in the presence of serum proteins and blood cells. Therefore, NGs were incubated with FBS at 37 °C over a period of 48 h. After distinct time points, samples were withdrawn from the solution and purified by fast protein liquid chromatography (FPLC) to separate the NGs from the serum proteins (Figure 3a). The NG stability was investigated by calculating the NG to FBS peak ratio, with a reduction in the NG peak integral suggesting partial NG degradation. From the chromatograms, after 24 and 48 h, NG peak ratios could be determined, ranging from 99 to 105%. This could indicate that parts of the serum proteins overlap with the NG peak or that some proteins adhere to the NG’s surface, leading to a simultaneous elution from the column. To clarify this observation, isolated NG samples were subjected to DLS analysis (Figure 3b). Here, no mixed fractions could be observed. Upon incubation with FBS, a slight reduction in the hydrodynamic diameter from 215 to 189 nm could be detected initially. However, there were no further changes in size over a period of 48 h. Moreover, zeta potential measurements revealed that the NGs kept their slightly negative surface charge, indicating the absence of charged serum proteins in the collected NG fraction (Table 2). The biocompatibility of the NGs was further assessed by conducting ex vivo hemolysis assays. Here, red blood cells were incubated with empty and loaded NGs, and the amount of released hemoglobin was detected spectrophotometrically to quantify red blood cell disruption [38]. Over a period of 24 h, less than 5% hemolysis could be detected for all tested NG compounds (Figure S3). Together with the results obtained from FBS stability studies, this indicates the biocompatibility of the NGs. 

### 2.5. Cellular Uptake

**Figure 4:**
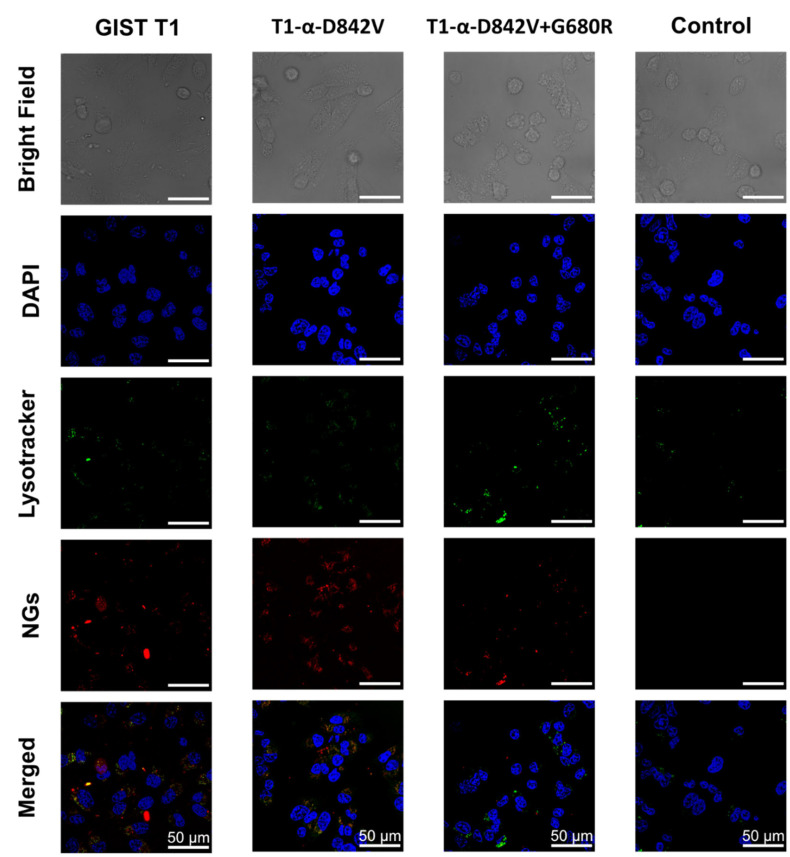
CLSM of GIST T1, T1-α-D842V, and T1-α-D842V-G680R cells with and without Cy5-labeled NGs (2 h incubation with 500 μg/mL). The strong fluorescent signal of covalently attached Cy5 (red) shows successful internalization of the NGs into the cells. Scale: bar 50 μm, blue: Hoechst (nuclei), green: Lysotracker (lyso- and endosomes), red: Cy5-labeled NGs.

To ensure the intracellular release of 17-AAG and BLU-285, it is necessary that the NGs can surpass the cellular membrane of GIST cells. To investigate this requirement, norbornene-NH_2_ was coupled to a Cy5 derivative via amide coupling (Figure S8) and covalently attached to dPG-O-metTet via iEDDA cyclization prior to NG formation (Figure S14). Then, GIST cells from the T1 family were exposed to the dye-labeled NGs over a period of 120 min, and confocal laser scanning microscopy (CLSM) was performed to demonstrate successful cell internalization (Figure 4). The NGs were again exposed to the GIST cells to quantify the kinetics behind the cellular uptake. After defined time points, the cells were rinsed, detached, and subjected to flow cytometry analysis to determine the number of Cy5-NG-containing cells. Over a period of 120 min, an increasing linear trend of Cy5 positive cells could be observed for all cell lines with comparable slopes (Figures S4 and S5). This suggests that the uptake of the NGs is not influenced by the genetic mutations of the GIST cells.

### 2.6. Cell Viability Studies

By proving the capability of GIST cell internalization and compatibility with serum proteins and red blood cells, cell viability measurements were performed to further investigate the in vitro properties of the empty and loaded NGs. 

**Figure 5:**
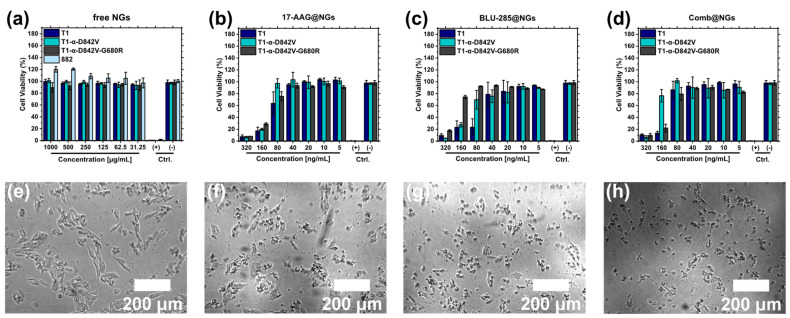
Cell viability of empty and loaded NGs. Cell viability of (**a**) empty NGs, (**b**) 17-AAG@NGs, (**c**) BLU-285@NGs, and (**d**) Comb@NG on GIST T1, T1-α-D842V, and T1-α-D842V-G680R cells determined by a CCK-8 assay. Live cell images of GIST-T1 cells after 48 h of treatment with (**e**) empty NGs, (**f**) 17-AAG@NGs, (**g**) BLU-285@NGs, and (**h**) Comb@NGs. Scale bar: 200 μm.

The empty NGs did not show a cytotoxic effect on the tested cell lines over a period of 48 h up to a concentration of 1000 μg/mL (Figure 5a). Exposing the cells to the drug-loaded analogs led to similar cytotoxic effects as the treatment with the free drugs in the range of 320 to 80 ng/mL (Figure 5b–d, Figure S6). As a visual proof for this observation, live cell imaging was performed with the T1 cell line and the NG compounds over a period of 48 h. While the presence of empty NGs did not influence cell proliferation and morphology, cell apoptosis could be observed in all cases for the loaded derivatives due to the formation of round and detached cells (Figure 5e–h). The obtained cell viability data were further analyzed by grating dose–response curves to estimate IC_50_ values (Table 3). The IC_50_ values of 17-AAG@NGs were 2-fold higher than those of the free 17-AAG. In the case of BLU-285, the opposite observation could be made, as the BLU-285@NGs demonstrated better inhibitions with IC_50_ values that were reduced by more than 2 folds compared to the free drug. Surprisingly, the resistant T1-α-D842V-G680R responded slightly to BLU-285@NGs with an IC_50_ value of 400 nM. Applying the free combinational treatment of 17-AAG and BLU-285 displayed high IC_50_ values in the range of 370–450 nM. However, the loaded Comb@NGs showed IC_50_ values for the T1 and T1-α-D842V that are in accordance those of the with 17-AAG@NGs and BLU-285@NGs. In the case of the resistant T1-α-D842V-G680R, the Comb@NGs showed the lowest IC_50_ value for the tested NG compounds with 138 nM. These results suggest that the NGs are capable of cell internalization and subsequent intracellular drug release. Furthermore, the obtained results from the Comb@NGs suggest that this system could help to address several GIST T1 tumors with varying genetic mutation patterns. 

## 3. Discussion

This work demonstrates the synthesis and detailed characterization of a novel esterase-responsive polyglycerol-based NG system that is formed by iEDDA-based crosslinking. Due to the hydrophobic segments in the NG framework, internalization of the hydrophobic drugs could be achieved through in situ encapsulation of BLU-285 and 17-AAG during inverse nanoprecipitation. The cleavage reaction of incorporated esters in the NG network structure was studied by ^1^H NMR spectroscopy and DLS measurements. It could be shown that hydrolysis rates were significantly enhanced upon CALB incubation compared to the PBS control, and NG degradation could be associated with ester cleavage. Biocompatibility of the NGs was proven by performing FBS stability studies and hemolysis assays. CLSM and flow cytometry revealed internalization of the NGs into various GIST T1 cell lines with comparable uptake kinetics. Cell viability measurements and live cell imaging showed that exposing different cells of the T1 family to the loaded NGs led to reduced cell viability, indicating intracellular drug release. Furthermore, it could be shown that multi-drug-loaded NGs showed a significant effect against the tested cell lines and induced apoptosis to BLU-285-resistant T1-α-D842V-G680R with comparably low IC_50_ values. This suggests that the present system could be a promising carrier platform for addressing multi-drug resistance in rare GISTs. Further studies, such as applying a tumor spheroid model, could help to reveal the full potential of the presented system. 

## 4. Materials and Methods

### 4.1. Chemicals and Reagents

5-Norbornene-2-carboxylic acid, DMF, pyridine, DMAP, Novozyme 435, Amphotericin B, *L*-Glutamine, HATU, and fetal bovine serum (FBS) were purchased from Sigma-Aldrich (Merck KGaA, Darmstadt, Germany). EDC·HCl was purchased from Carl-Roth (Carl Roth GmbH + Co. KG, Karlsruhe, Germany). BLU-285 and 17-AAG were purchased from MedChemExpress (MedChemExpress EU—MedChemTronica, Sollentuna, Sweden). Penicillin-Streptomycin (Pen-Strep) was purchased from Invitrogen. Iscove’s Modified Dulbecco’s Medium (IMDM) and LysotrackerTM Green DnD-26 were purchased from Thermo Fisher Scientific (Thermo Fisher Scientific Inc., Darmstadt, Germany). Water was used from a Milli-Q station from Millipore. All reactions were performed under an argon atmosphere using standard Schlenck techniques and an oil pump vacuum.

### 4.2. NMR

In the described deuterated solvents, ^1^H-NMR spectra were obtained at 300 K on a Joel ECX 400 (400 MHz) or a Bruker Avance III (700 MHz). Chemical shifts (δ) are reported in parts per million (ppm) with regard to the corresponding solvent peaks. A Bruker Avance III (176 MHz) was used to record ^13^C-NMR at 300 K. Chemical shifts are expressed in parts per million (ppm) with respect to the residual solvent peaks. The spectra were decoupled from proton broadband.

### 4.3. GPC

Gel permeation chromatography (GPC) was measured on an AGILENT 1100 at 5 mg/mL sample concentration using a pullulan standard 0.1 M NaNO_3_ solution as an eluant and a PSS Suprema column 10 μm with a flow rate of 1 mL/min. The signals were detected with an RI detector.

### 4.4. Synthesis of Materials

**Synthesis of metTet-COOH, Cy5-Norb, and dPG-OH**

metTet-COOH and dPG-OH were synthesized according to the literature [39,40] (Figures S7–S9).

**Synthesis of dPG-O-metTet**

metTet-COOH (1.11 g, 5.15 mmol, 1.2 eq.) was dissolved in dry DMF (40 mL). HATU (2.12 g, 5.58 mmol, 1.3 eq.), DMAP (0.68 g, 5.58 mmol, 1.3 eq.), and EDC·HCl (0.99 g, 5.15 mmol, 1.2 eq.) were added. The solution was stirred for 1.5 h at rt. In a second Schlenk flask, dPG (3.97 g, 0.397 mmol, 1 eq.) was dissolved in dry DMF (20 mL), and DMAP (0.68 g, 5.58 mmol, 1.3 eq.) and pyridine (0.41 g, 5.15 mmol, 1.2 eq.) were added. The solution containing the 5-Norbornene-2-carboxylic acid was added to the polymer solution for 30 min via a syringe. The solution was stirred overnight at 60 °C and purified with dialysis in methanol for 4 days. dPG-O-Met-Tet was obtained as a purple methanolic solution (1.05 g, 4.89 mmol, dF = 8%, 95%). ^1^H NMR (700 MHz, CD_3_OD): δ = 8.70–8.36 (m, 2 H, H-aryl), 8.34–8.00 (m, 2 H, H-aryl), 4.03–3.38 (m, dPG-backbone), and 3.07 (s, 3 H, methyl-H) ppm (Figure S10). ^13^C NMR (176 MHz, CD_3_OD): δ = 169.2, 167.1, 164.7, 137.6, 134.6, 131.5, 128.9, 81.6, 81.4, 80.1, 79.9, 74.0, 73.0, 72.5, 72.4, 72.2, 71.0, 70.7, 69.8, 69.6, 67.8, 66.2, 64.5, 64.4, 62.8, 61.9, 49.9, 49.4, 49.2, 49.1, 49.0, 48.9, 48.8, 48.6, and 21.4 ppm (Figure S11).

**Synthesis of dPG-O-Norb**

5-Norbornene-2-carboxylic acid (1.84 g, 13.33 mmol, 1.2 eq.) was dissolved in dry DMF (50 mL). HATU (5.49 g, 14.44 mmol, 1.3 eq.), DMAP (1.76 g, 14.44 mmol, 1.3 eq.), and EDC·HCl (2,56 g, 13.33 mmol, 1.2 eq.) were added, and the solution was stirred for 1.5 h at rt. In a second Schlenk flask, dPG (5.48 g, 0.548 mmol, 1 eq.) was dissolved in dry DMF (15 mL), and DMAP (1.76 g, 14.44 mmol, 1.3 eq.) and pyridine (1.05 g, 13.33 mmol, 1.2 eq.) were added. The 5-Norbornene-2-carboxylic acid solution was added to the polymer solution for 30 min via a syringe. The resulting solution was stirred overnight at 60 °C and purified with dialysis in methanol for 4 days. dPG-O-Norb was obtained as a colorless methanolic solution (1.58 g, 11.46 mmol, dF = 6.0%, 86%).

^1^H NMR (700 MHz, CD_3_OD): δ = 8.70–8.40 (m, 1 H, H-olefin), 8.35–8.31 (m, 1 H, H-olefin), 4.00–3.40 (m, dPG-backbone), 3.28–3.14 (m, 1 H, H-bridgehead), 3.12–2.98 (m, 1 H, H-bridgehead), 2.95–2.18 (m, 1 H, H-bridgehead), and 1.60–0.85 (m, 1 H, H-bridge) ppm (Figure S12).

^13^C NMR (176 MHz, CD_3_OD): δ = 169.2, 167.1, 164.7, 137.6, 134.6, 131.5, 128.9, 81.6, 81.4, 80.1, 79.9, 74.0, 73.0, 72.5, 72.4, 72.2, 71.0, 70.7, 69.8, 69.6, 67.8, 66.2, 64.5, 64.4, 62.8, 61.9, 49.9, 49.4, 49.2, 49.1, 49.0, 48.9, 48.8, 48.6, and 21.4.ppm (Figure S13).

**Cy5-Labeling of dPG-O-metTet**

Cy5-Norb (8.00 mg, 0.01 mmol) was added to a solution of dPG-O-metTet (50 mg, 5.00 nmol) in water (1.00 mL), heated to 37 °C, and stirred for 4 h. The obtained product was purified via SEC (Sephadex^TM^ G-25) to obtain the Cy5-labeled dPG-O-metTet (13 mg/mL in water) (Figure S14).

**Cy5-Labeling of NGs**

Cy5-dPG-O-metTet (0.4 mg, 30 μL from 13 mg/mL stock) and dPG-O-metTet (2.6 mg, 13 μL from 200 mg/mL stock) were diluted in water (467 μL). In a separate vial, dPG-O-Norb (3.00 mg, 15 μL from 200 mg/mL stock) was diluted in water (485 μL). The resulting dPG-O-Norb solution was added quickly to the dPG-O-metTet solution, briefly vortexed for 5 s, and the combined mixture was injected into acetone (20 mL) under vigorous stirring. The suspension was stirred for another 5 s and then kept still. After 30 min, water (10 mL) was added to terminate the reaction. The solution was transferred into a 50 mL round bottom flask, and the solvent was evaporated under reduced pressure to yield NGs with a final concentration of 5 mg/mL. Purification and transfer to a PBS solution were achieved through dialysis (MWCO = 100 kDa, 4 days).

### 4.5. Procedures

#### 4.5.1. NG Formation

The NGs were prepared through inverse nanoprecipitation following a protocol in the literature [39].

Macromonomers dPG-O-metTet and dPG-O-Norb were stored in aqueous stock solutions (200 mg/mL, MilliQ-water). A total of 53 μL of dPG-O-metTet (8.00 mg, 40 μL from 200 mg/mL stock) was diluted in water (460 μL). In a separate vial, 80 μL of the dPG-O-Norb (12 mg, 60 μL from 200 mg/mL stock) was diluted with water (480 μL). The resulting dPG-O-Norb solution was added quickly to the dPG-O-metTet solution, briefly vortexed for 5 s, and the combined mixture was injected into acetone (20 mL) under vigorous stirring. The suspension was stirred for another 5 s and then kept still. After 20 min, water (10 mL) was added to terminate the reaction. The solution was transferred into a 50 mL round bottom flask, and the solvent was evaporated under reduced pressure to yield NGs with a final concentration of 20 mg/mL. Purification and transfer to a PBS solution were achieved through dialysis (MWCO = 100 kDa, 4 days).

#### 4.5.2. Cryo-TEM

At room temperature, 4 μL of the NG solution were placed on hydrophilized holey carbon-filmed grids (Quantifoil R1/2), while the excess fluid was removed by utilizing filter paper to create an ultra-thin solution layer that spans the holes in the carbon film. After vitrification in liquid ethane, which was performed with an automated vitrification robot (FEI Vitrobot Mark III), the grids were stored in liquid nitrogen. They were stabilized by a copper auto grid and fixed with a spring clamp under liquid nitrogen. The auto grids were transferred into the microscope under liquid nitrogen using the microscope’s autoloader. Cryogenic transmission electron microscopy (cryo-TEM) was performed using a Talos Arctica transmission electron microscope (Thermo Fisher Scientific Inc., Waltham, MA, USA. Micrographs were obtained by following the microscope low-dose protocol at 28,000× primary magnification and 200 kV acceleration voltage. Images were captured at full resolution (4k) using a Falcon 3CE direct electron detector (48 aligned frames). An appropriate contrast was achieved by setting the de-focus to 5 μm.

#### 4.5.3. Drug Encapsulation

The loading of 17-AAG, BLU-285, and their combination was achieved through coprecipitation. 17-AAG and BLU-285 were stored in a sock solution (20 mg/mL, DMSO). The desired drug (1.00 mg, 50 μL from 20 mg/mL stock) was added to the diluted dPG-O-metTet solution, and the NG formation protocol was carried out. The drug loading content (*DLC*) was determined via UV/Vis for 17-AAG using its unique absorbance at 550 nm (Figure S9**)** and via fluorescence for BLU-285 using 380 nm as λ_ex_ and 440 nm as λ_em_ (Figure S10) and calculated via Equation (1). The encapsulation efficiency (ee) was calculated as depicted in Equation (2) by dividing the determined amounts of encapsulated drugs (*DLC_determined_*) by the drug amounts used encapsulation *DLC_theory_*_._ UV/Vis measurements were conducted on an Agilent Cary 8454 UV-visible spectrophotometer using half-micro quartz cuvettes. Fluorescence spectroscopy was performed on a Tecan plate reader (infinite pro200, TECAN-reader Tecan Group Ltd., Zurich, Switzerland).

(1) $$DLC\;\left(wt\%\right)=\frac{m_{loaded\;drug}}{m_{NG}}$$

(2) $$ee\;\left(\%\right)=\frac{{DLC}_{determined}}{{DLC}_{theory}}*100$$

#### 4.5.4. Macromonomer Degradation

dPG-O-Norb (2 × 30 mg) was taken from the methanolic stock solutions and dried overnight. Each sample was dissolved in a deuterated PB buffer solution (1.00 mL, 10 mM, pH = 7.4), and 200 wt% of Novozyme 435 with respect to the polymer amount was added to one of the samples. DMSO (10 Vol%, 0.10 mL) was added to the buffer solution to prevent precipitation of the formed degradation products in water, and the pH was again adjusted to 7.4. Afterward, all NMR tubes were incubated for 1 h at 37 °C and measured at 400 MHz. After each measurement, the samples were stored in the incubator at 37 °C until the next measurement cycle started.

#### 4.5.5. NG Degradation

Degradation of the obtained NGs was determined through DLS measurements. Two samples of NGs were diluted to a final concentration of 5 mg/mL in PBS. A total of 200 wt % of Novozyme 435 with respect to the NG amount was added to one of the samples. They were incubated at 37 °C, and DLS measurements were conducted each day to determine the hydrodynamic diameter of the NGs. All measurements were conducted in triplicates. Dynamic light scattering (DLS) and zeta potential were measured on a Malvern zeta-sizer nano ZS ZEN 3600 using a He-Ne laser (λ = 532 nm) at 173° backscatter and automated attenuation at, if not stated differently, 37 °C. All sample measurements were performed as triplicates, yielding a mean size value plus standard deviation.

#### 4.5.6. Serum Stability

The obtained NGs (0.1 mL from 20 mg/mL stock) were diluted in fetal bovine serum (FBS) (1 mL) and incubated at 37 °C. After 0 h, 24 h, and 48 h, 0.3 mL of the solution was withdrawn and purified via fast protein liquid chromatography. The collected NG fractions were further analyzed in DLS. The stability of the NGs in the presence of FBS was determined by dividing the peak area of the NGs by the peak area of the FBS. This quotient was set to 100% for the 0 h measurement. In all further measurements (*i* = 24 h, 48 h), the stability was determined in relation to this quotient (Equation (3)). All measurements were performed as triplicates. Fast protein liquid chromatography was conducted on an ÄKTAprime plus device (GE Lifescience, Chicago, IL, USA) equipped with a Superdex 200 10/300 GL column, UV detector (280 nm), and 100 μL loop. PBS (154 mM, pH 7.4) was used as an eluent. All measurements were conducted with a flow rate of 0.7 mL/min.

(3) $$Stability\;\left(\%\right)=100*\;\frac{\frac{{Peak\;Area\;of\;NG}_{i}}{{Peak\;Area\;of\;FBS+NG}_{i}}}{\frac{{Peak\;Area\;of\;NG}_{0\;h}}{{Peak\;Area\;of\;FBS+NG}_{0\;h}}}$$

*i* = 0, 1, 2 days.

#### 4.5.7. Ex vivo Red Blood Cell Hemolysis Assay

The hemocompatibility of free NGs and their loaded analogs (17AAG@NGs, BLU-285@NGs, and comb@NGs) was evaluated according to a published protocol [38]. Briefly, fresh donor blood was separated into serum and red blood cells. After washing, a 1:25 dilution of erythrocytes (100 µL) was incubated at 37 °C for 1 h, 4 h, and 24 h with different concentrations of the abovementioned samples (100 µL). DPBS (100 µL) was used as the negative control and 1% Triton X-100 (100 µL) as the positive control. The supernatant (100 µL) was transferred to a flat 96-well plate for absorbance measurements at 410 nm. Absorption is proportional to hemoglobin content in the supernatant. This allows conclusions to be drawn about the effect of a compound on cell lysis. Results are plotted as % hemolysis after subtraction of the absorbance of the negative control and normalization to the positive control (Figure S11).

#### 4.5.8. Cell Lines and Cultivation

All cell lines were kindly gifted by Prof. Sebastian Bauer from UK Essen. GIST882 cells were cultured in RPMI 1640 supplemented with 15% (*v*/*v*) of FBS, a 1% (*v*/*v*) penicillin–streptomycin solution (100 IU/mL of Penicillin G Sodium Salt and streptomycin sulfate (100 µg/mL)) (Pen-Strep), and 1% (*v*/*v*) l-glutamine. The remaining cell lines were grown in IMDM supplemented with 10% (*v*/*v*) of FBS, a 1% (*v*/*v*) penicillin–streptomycin solution, and 1% (*v*/*v*) l-glutamine. T1-α-D842V and T1-α-D842V + G680R cells were treated with Imatinib (200 nM) and BLU-285 (100 nM) drugs, respectively.

#### 4.5.9. Cellular Uptake Studies

CLSM monitored Cellular Uptake. A total of 20,000 cells per well were seeded on 8-well ibidi slides and incubated for 48 h. Then, the cells were washed with PBS and treated with Cy5-labeled NGs with a final concentration of 500 μg/mL. After 2 h of incubation, the cells were stained with Hoechst 33,342 (1 μg/mL), washed twice with PBS, covered with a fresh cell culture medium, and incubated with Lysotracker Green DND 26 (500 nM) for 10 min before imaging. Confocal pictures were captured using an inverted confocal laser scanning microscope, Leica DMI6000CSB SP8 (Leica, Wetzlar, Germany), with a 63×/1.4 HC PL APO CS2 oil immersion objective, as well as the manufacturer-supplied LAS X 5.1.0 software.

The kinetics of NG uptake into the GIST cells was determined by flow cytometry. GIST cells were seeded on a 24-well plate at a concentration of 150,000 cells per well. After 48–72 h of seeding, cells were treated with Cy5-labeled NGs with a final concentration of 500 μg/mL Cy5 in 0.5 mL medium for 15, 30, 60, and 120 min. After the treatment, the cells were rinsed twice with PBS and detached using 200–250 μL trypsin. The cells were collected with medium, centrifuged at 400× *g* for 5 min, and resuspended in fresh medium. The cell suspension was subjected to flow cytometry analysis (Figures S12 and S13). Flow cytometry was performed on a Atune NxT (Thermo Fisher) flow cytometer. Data were acquired by Attune™ NxT Software v2.7 and further processed with FlowJo V10 analysis software.

#### 4.5.10. Cell Viability

Cell viability of the loaded and free NGs on GIST T1, GIST 882, T1-α-D842V, and T1-α-D842V + G680R cells was determined by following a protocol in the literature for the CCK-8 assay [39]. In a 96-well plate, 90 μL of PBS was added to each outer well. For the inner wells, 90 μL of a cell suspension containing 5 × 10^4^ cells were seeded into each well, and the plate was incubated at 37 °C and 5% CO_2_ overnight. For the tested compounds, serial dilutions were performed to obtain the desired concentrations, and 10 μL of each was added to the cells in triplicates. SDS (1%) and non-treated cells were used as positive and negative controls, respectively. After 42 h of incubation at 37 °C and 5% CO_2,_ 10 μL of the CCK-8 solution was added to each well. After another 3 h of incubation, the absorbance at 450 nm was measured in triplicates by utilizing a Tecan plate reader (Infinite Pro200, TECAN-reader Tecan Group Ltd.). The absorbance at 650 nm was used as an internal reference wavelength. Cell viability was determined by setting the obtained values for the non-treated control to 100% after subtracting the background using Microsoft Excel^®^ 2019 software. All other values were calculated in relation to this value following the same procedure. The half-maximal inhibitory concentration (IC50) was calculated with GraphPad Prism 6.01 (Graph Pad Software, San Diego, CA, USA) using the log (inhibitor) vs. normalized response variable slope equation. Lionheart FX Automated Live Cell Image (Bio Tek Instruments, Winooski, VT, USA) was used to examine the effect of empty and loaded NGs on the morphology of T1 cells. A high-contrast brightfield accessory kit (Bio Tek, Winooski, VT, USA) was used to monitor the cellular morphology changes. Images were captured at 4× in a high-contrast brightfield channel. A total of 10,000 cells per well were seeded on a 96-well culture plate for 24 h. The cells were then treated with NGs (100 μL) and live-imaged for 48 h. Environmental conditions were maintained at 37 °C and 5% CO_2_ within the Lionheart throughout the imaging.

All cell experiments were conducted according to German genetic engineering laws and German biosafety guidelines in the laboratory (safety level 1).

## Figures and Tables

**Table 1:** The table shows obtained data from loading experiments with 17-AAG and BLU-285 where [wt%]_theo_ specifies the drug amounts used for drug for encapsulation, and [wt%]_calc_ the drug amounts determined after encapsulation and purification.

Entry	Name		DLC [wt%]_theo_	DLC [wt%]_calc_^1^	ee (%)	Size [d.nm] ^2^	PDI ^2^	ζ—Potential [mV] ^2^
1	Empty NGs		-	-	-	257 ± 7	0.06 ± 0.01	−0.6
2	17-AAG@NGs		5	4.95	99	276 ± 4	0.20 ± 0.01	1.9
3	BLU-285@NGs		5	4.95	99	272 ± 12	0.10 ± 0.04	3.9
4	Comb@NGs	17-AAG	5	5.00	Quant.	361 ± 18	0.21 ± 0.05	3.8
		BLU-285	5	3.10	62			

^1^ Determined via UV/Fluorescence λ_abs_ = 550 nm for 17-AAG; λ_ex_ = 380 nm, λ_abs_ = 440 nm at 25 °C in 1.5 mM PBS. ^2^ Determined with a Zetasizer Nano-ZS at 25 °C in 1.5 mM PBS.

**Table 2:** The table shows analysis data of FBS stability studies obtained from chromatograms of Figure 3a and DLS measurements of Figure 3b.

Time	NG Area (%) ^1^	Size [d.nm] ^2^	PDI ^2^	Ζ—Potential [mV]^2^
Prior to inc.	-	215 ± 1	0.10 ± 0.10	−0.6 ± 0.5
0 day	100.00 ± 0.71	189 ± 3	0.03 ± 0.06	−2.26 ± 0.5
1 day	104.48 ± 0.67	198 ± 4	0.10 ± 0.02	−2.08 ± 0.4
2 days	99.77 ± 0.74	182 ± 2	0.15 ± 0.02	−1.94 ± 0.7

^1^ Determined from FPLC chromatograms by dividing the NG peak area by the FBS peak area. ^2^ Measured with Zetasizer Nano-ZS at 25 °C in 1.5 mM PBS.

**Table 3:** Determined IC_50_ values from Figure 5b–c and Figure S6.

Cell Line	IC50 [nM]					
	Free 17-AAG	17-AAG@NGs	Free BLU-285	BLU-285@NGs	Free Comb	Comb@NGs
GIST T1	89 ± 1	165 ± 1	446 ± 2	120 ± 1	451 ± 3	136 ± 1
T1-α-D842V	104 ± 1	224 ± 1	383 ± 3	190 ± 2	377 ± 3	236 ± 2
T1-α-D842V-G680R	104 ± 1	236 ± 1	-	401 ± 3	370 ± 3	138± 2

## Data Availability

Data is contained in the paper.

## Supplementary Materials

The following supporting information can be downloaded at https://www.mdpi.com/article/10.3390/ph16111618/s1, Figure S1: Calibration curve of 17-AAG obtained from UV/Vis measurements at 550 nm; Figure S2: Calibration curve of BLU-285 obtained from fluorescence measurements using 380 nm as λ_ex_ and 440 nm as λ_em_; Figure S3: Results of the ex vivo red blood cell hemolysis assay. The absorbance of the supernatant at 410 nm after the ex vivo red blood cell hemolysis assay of a) empty NGs and b) loaded NGs (n = 3) after 24 h. DPBS was used as a negative control ((−), n = 6) and 1% Triton X-100 as a positive control ((+), n = 6); Figure S4: Flow cytometry histograms of (a) GIST T1, (b) T1-α-D842V, and (c) T1-α-D842V-G680R cells treated with Cy5-labeled NGs over a period of 48 h; Figure S5: Fluorescence intensity quantification of flow cytometry experiments; Figure S6: Cell viability results of (a) free 17-AAG, (b) free BLU-285, and (c) free comb; Figure S7: ^1^H NMR (400 MHz, DMF-d7) of metTet-COOH; Figure S8: ^1^H NMR (700 MHz, CD_3_OD) of Cy5-Norb; Figure S9: GPC of dPG-OH; Figure S10: ^1^H NMR (700 MHz, CD_3_OD) of dPG-O-metTet; Figure S11: ^13^C NMR (176 MHz, CD_3_OD) of dPG-O-metTet; Figure S12: ^1^H NMR (700 MHz, CD_3_OD) of dPG-O-Norb; Figure S13: ^13^C NMR (176 MHz, CD_3_OD) of dPG-O-Norb; and Figure S14: UV/Vis spectra of dPG-O-metTet (blue) and Cy5-dPG-O-metTet (green).
