# Supplementary material for: Esterase-Responsive Polyglycerol-Based Nanogels for Intracellular Drug Delivery in Rare Gastrointestinal Stromal Tumors
Source: Pharmaceuticals (Basel). 2023 Nov 16;16(11):1618. doi: 10.3390/ph16111618 (PMC10675119; doi:10.3390/ph16111618)
Supplement: Supplementary file 1 [file pharmaceuticals-16-01618-s001.zip › pharmaceuticals-2687155-supplementary.pdf]

Esterase-Responsive Polyglycerol-Based Nanogels for Intracellular Drug Delivery in Rare Gastrointestinal Stromal Tumors

Sebastian Schötz[1], Adele K. Griepe[1], Björn B. Goerisch[1], Sally Kortam[2], Yael Shammai Vainer[3], Mathias Dimde[4], Hanna Koeppe[1], Stefanie Wedepohl[5], Elisa Quaas[5], Katharina Achazi[5], Avi Schroeder[3,*], Rainer Haag[1,5,*]

1 Institute of Chemistry and Biochemistry, Freie Universität Berlin, Takustr. 3, 14195 Berlin, Germany

2 School of Biomedical Engineering, The University of Sydney, NSW 2006, Australia

3 The Louis Family Laboratory for Targeted Drug Delivery and Personalized Medicine Technologies, Technion, Haifa 32000, Israel

4 Research Center of Electron Microscopy, Freie Universität Berlin, Fabeckstr. 36A, 14195 Berlin, Germany

5 Research Building SupraFAB, Freie Universität Berlin, Altensteinstr. 23a, 14195 Berlin, Germany

* Corresponding Authors

Email: avids@technion.ac.il (A.S.); haag@chemie.fu-berlin.de (R.H.)

Keywords, Nanogels, Drug Delivery, GIST, iEDDA, Poly Glycerol

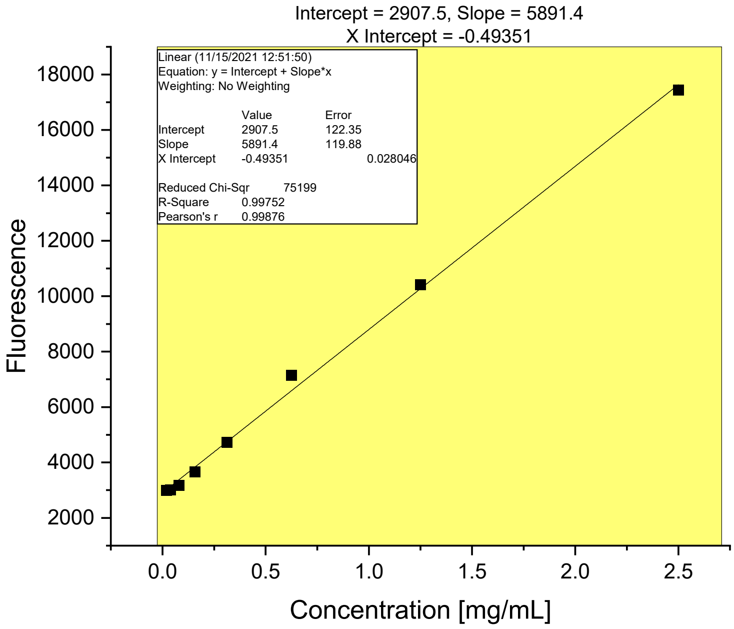

Figure S1: Calibration curve of 17-AAG obtained from UV/Vis measurements at 550 nm.

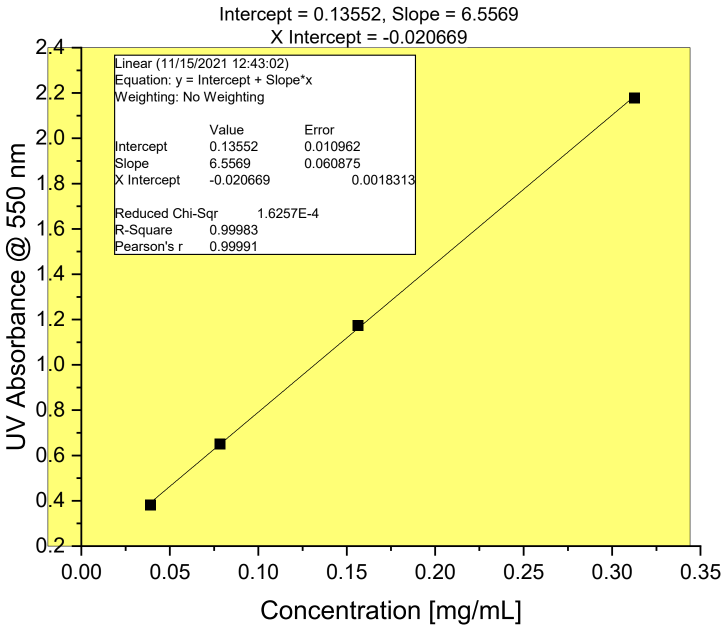

Figure S2: Calibration curve of BLU-285 obtained from fluorescence measurements using 380 nm as λ_{ex} and 440 nm as λ_{em}.

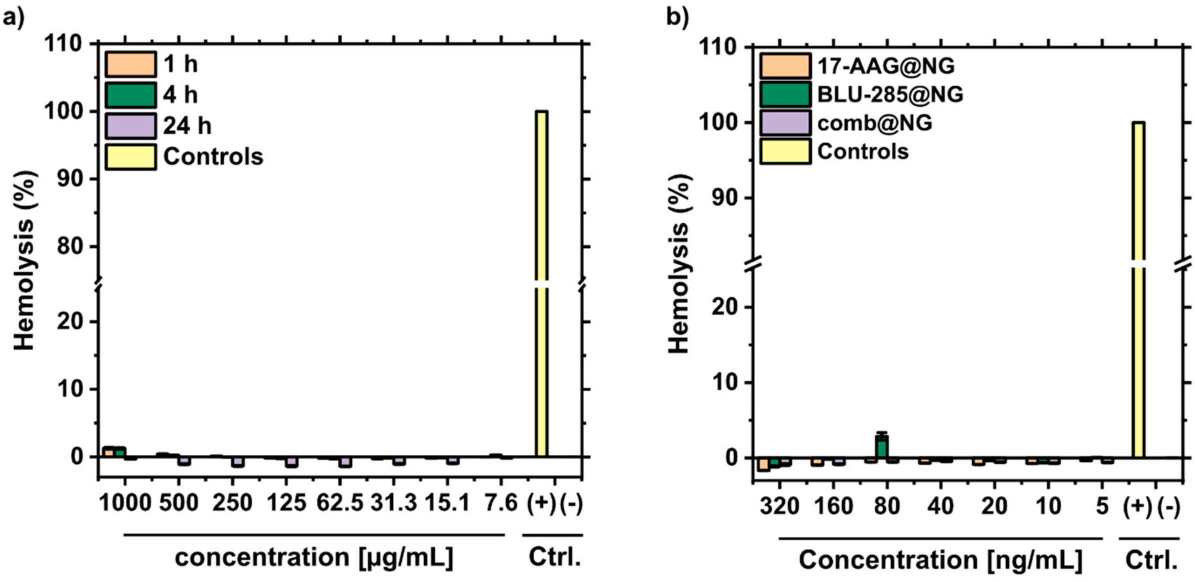

Figure S3: Results of ex vivo red blood cell hemolysis assay. Absorbance of supernatant at 410 nm after ex vivo red blood cell hemolysis assay of (a) empty NGs and (b) loaded NGs (n = 3) after 24 h. DPBS was used as negative control ((-), n = 6) 1% Triton X-100 as positive control ((+), n = 6).

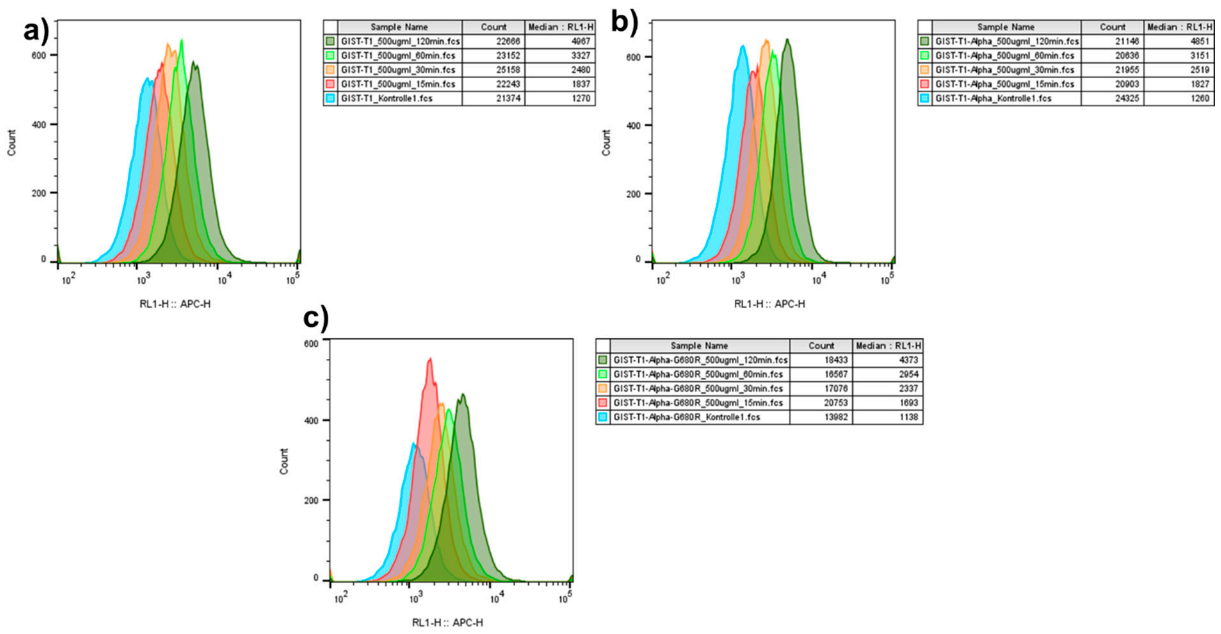

Figure S4: Flow cytometry histograms of (a) GIST T1, (b) T1-α-D842V and (c) T1-α-D842V-G680R cells treated with cy5-labeled NGs over a period of 48 h.

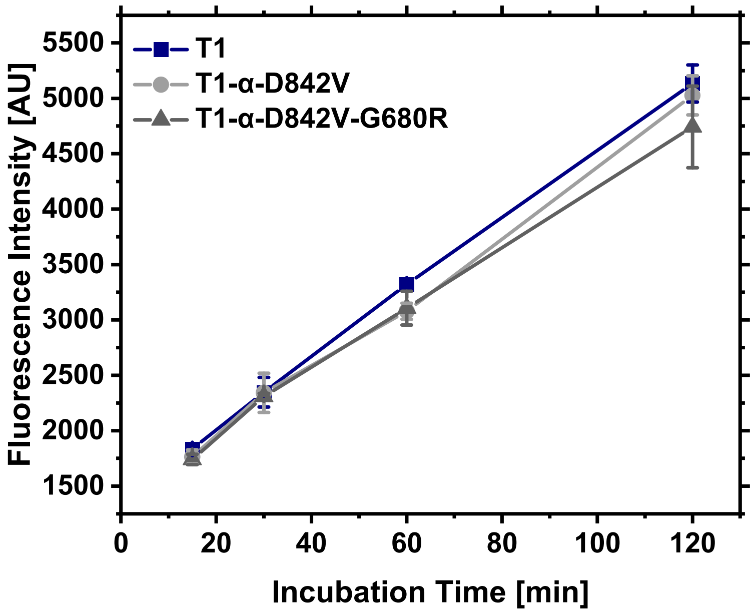

Figure S5: Fluorescence intensity quantification of flow cytometry experiments.

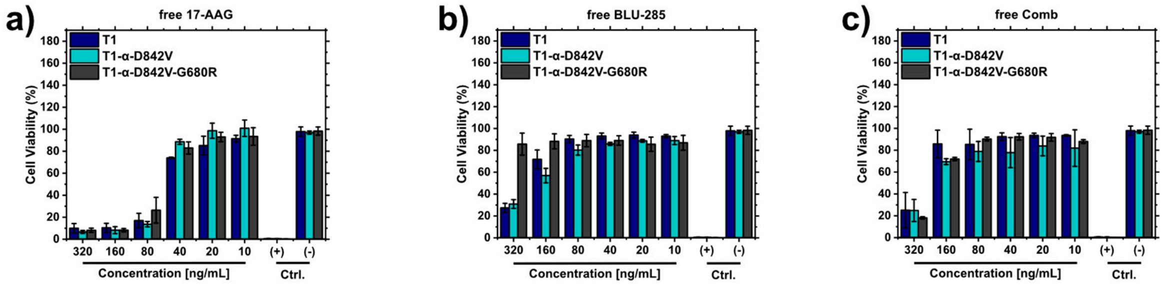

Figure S6: Cell viability results of (a) free 17-AAG, (b) free BLU-285 and (c) free comb.

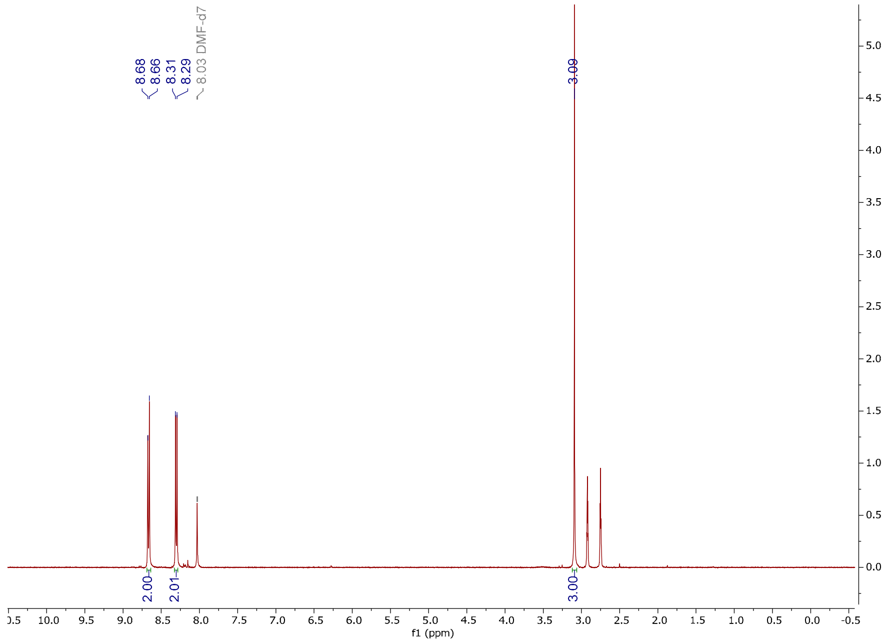

Figure S7: ^{1}H NMR (400 MHz, DMF-d7) of metTet-COOH.

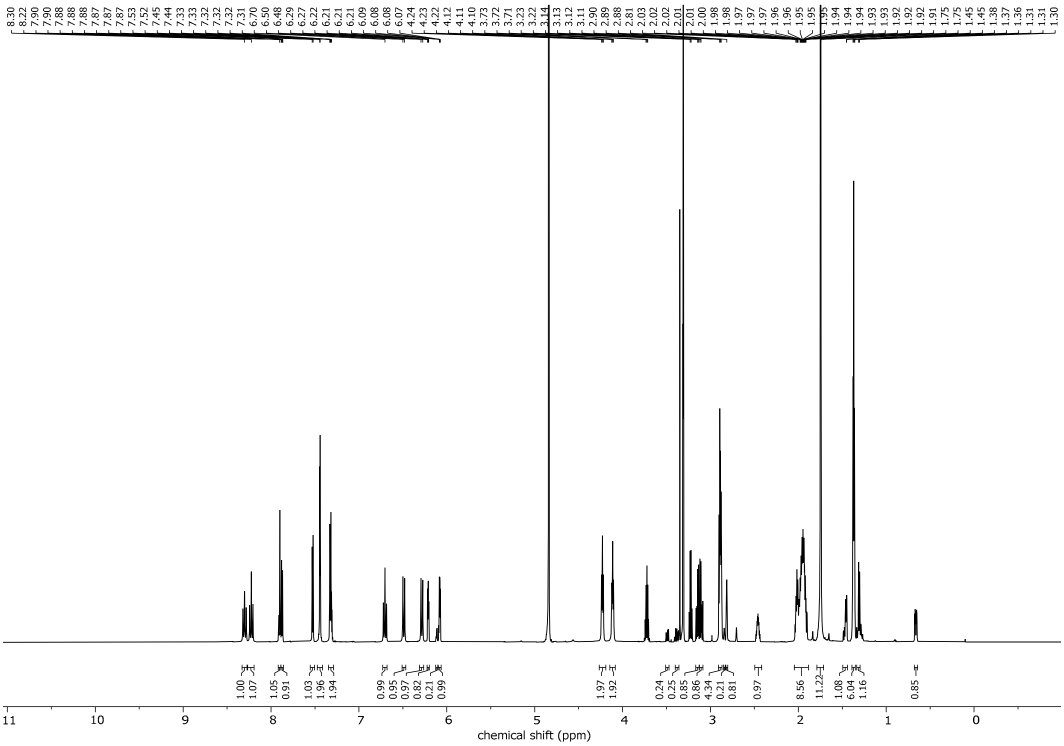

Figure S8: ^{1}H NMR (700 MHz, CD_3OD) of cy5-Norb.

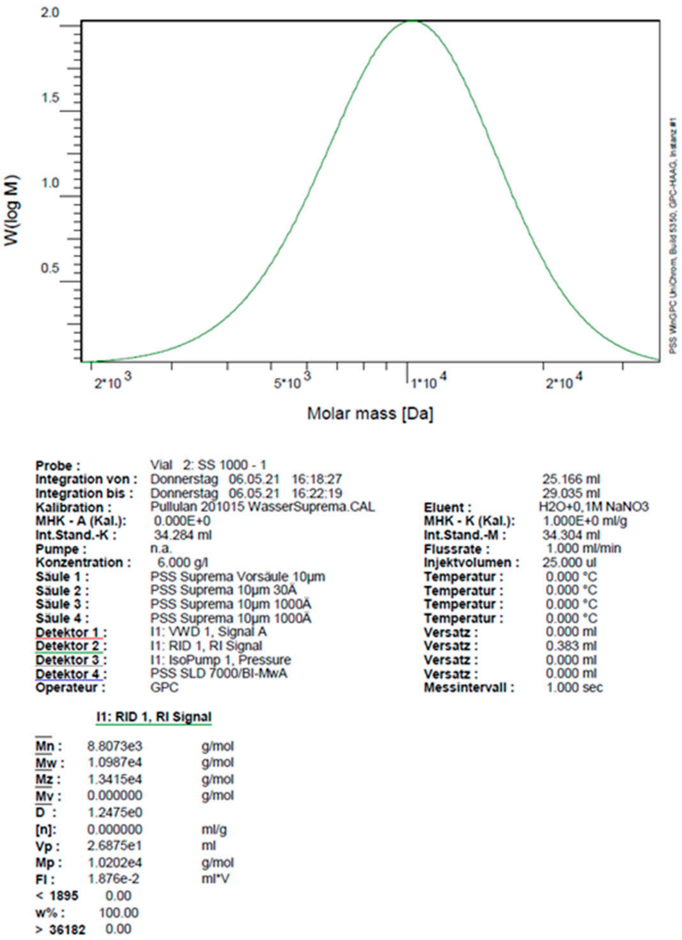

Figure S9: GPC of dPG-OH.

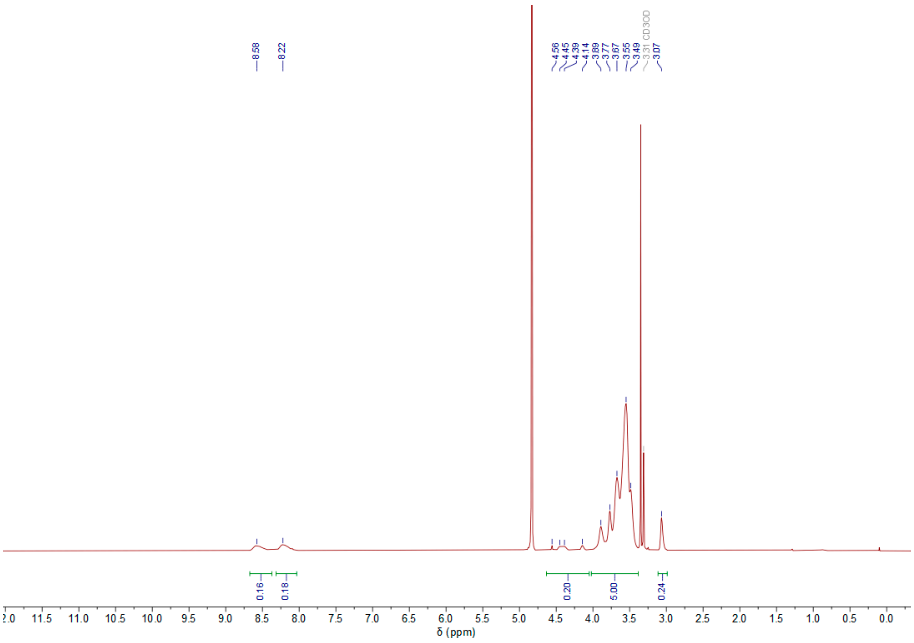

Figure S10: ^{1}H NMR (700 MHz, CD_3OD) of dPG-O-metTet.

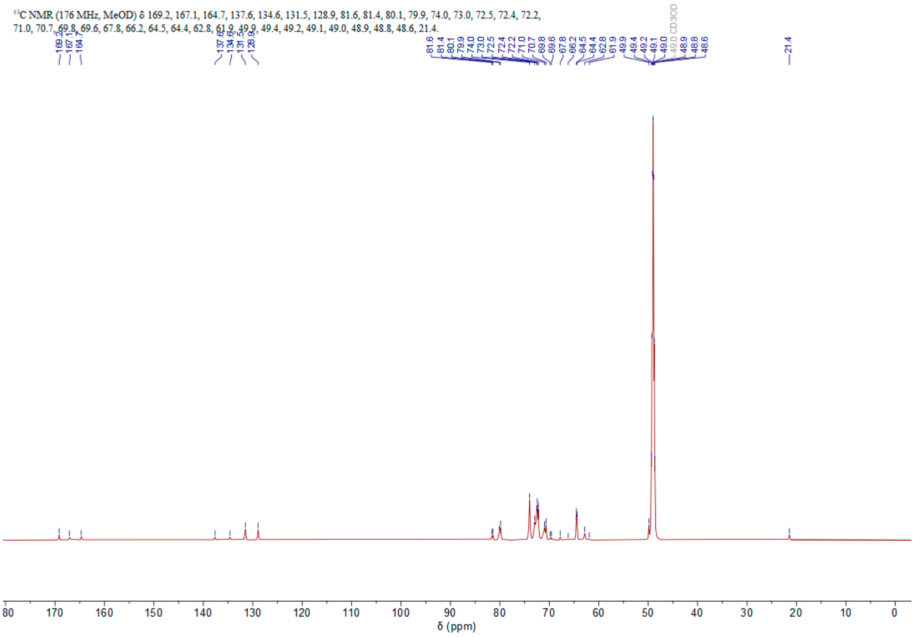

Figure S11: ^{13}C NMR (176 MHz, CD_3OD) of dPG-O-metTet.

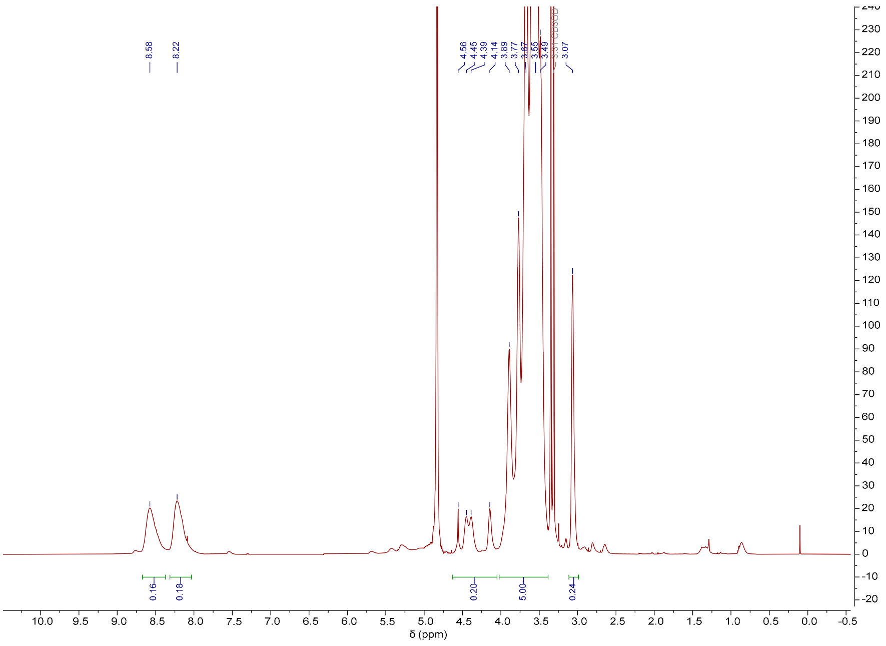

Figure S12: ^{1}H NMR (700 MHz, CD_3OD) of dPG-O-Norb.

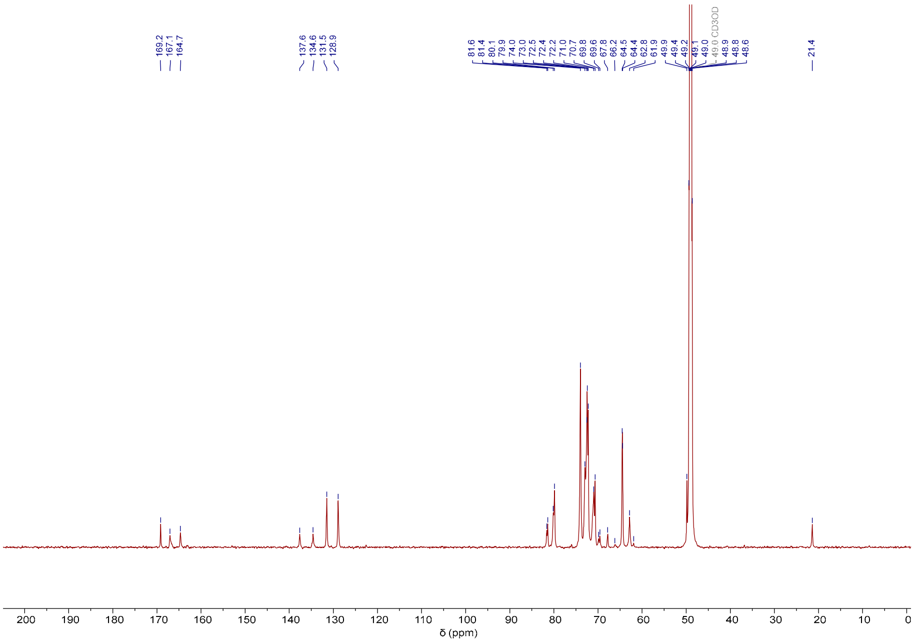

Figure S13: ^{13}C NMR (176 MHz, CD_3OD) of dPG-O-Norb.

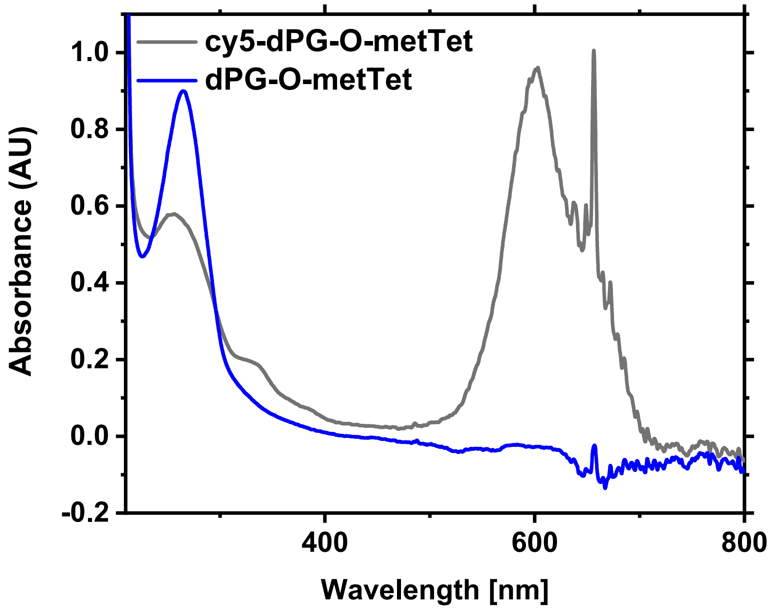

Figure S14: UV/Vis spectra of dPG-O-metTet (blue) and cy5-dPG-O-metTet (green).
